# LncRNA NEAT1 Enhances Glioma Progression via Regulating the miR-128-3p/ITGA5 Axis

**DOI:** 10.1007/s12035-021-02474-y

**Published:** 2021-07-15

**Authors:** Jiakai Chen, Handong Wang, Junjun Wang, Wenhao Niu, Chulei Deng, Mengliang Zhou

**Affiliations:** 1 Department of Neurosurgery, Jinling Hospital, Medical School of Nanjing University, 305 East Zhongshan Road, Nanjing, 210002 Jiangsu People’s Republic of China; 2 Department of Clinical Laboratory, Jinling Hospital, Medical School of Nanjing University, 305 East Zhongshan Road, Nanjing, 210002 Jiangsu People’s Republic of China; 3 Department of Neurosurgery, Jinling Hospital, Medical School of Southeast University, 305 East Zhongshan Road, 210002 Nanjing, Jiangsu People’s Republic of China; 4 Department of Neurosurgery, Jinling Hospital, the first School of Clinical Medicine, Southern Medical University, 305 East Zhongshan Road, Nanjing, 210002 Jiangsu People’s Republic of China

**Keywords:** NEAT1, miR-128-3p, ITGA5, Glioma, FAK

## Abstract

Accumulating evidences indicate that long non-coding RNA nuclear paraspeckle assembly transcript 1 (NEAT1) promotes the progression of glioma. In this study, we postulated that NEAT1 may act as a miR-128-3p sponge. Relative levels of NEAT1 and miR-128-3p expression in human glioma samples and GBM cells were detected using quantitative real-time PCR. By means of CCK-8 assays, transwell assays, and flow cytometric analysis, the biological functions of miR-128-3p and NEAT1 were investigated in U87MG and U251MG human GBM cell lines with stable miR-128-3p and NEAT1 knockdown or overexpression. The luciferase reports, RNA pull-down assay, and RNA immunoprecipitation assay were conducted to determine the relevance of NEAT1 and miR-128-3p in glioma. As a result, high expression of NEAT1 and lack of miR-128-3p were observed in glioma specimens and cells. By binding to anti-oncogene miR-128-3p in the nucleus, NEAT1 enhanced tumorigenesis and glioma development. Further experiments suggested that ITGA5 expression was increased in glioma tissues and was found to be connected with miR-128-3p. Additionally, NEAT1 facilitated ITGA5 expression via competitively binding to miR-128-3p. For this reason, ITGA5 would not be decomposed by miR-128-3p and could activate FAK signaling pathway, thereby promoting cell growth. Collectively, these results indicated that the NEAT1/miR-128-3p/ITGA5 axis was involved in glioma initiation and progression, and might offer a potential novel strategy for treatment of glioma.

## Introduction

Among the central nervous system (CNS), glioma is the most frequently occurring primary intraparenchymal tumor [1]. Although researchers have developed early diagnostic and treatment therapies, including surgery, chemotherapy, and radiotherapy, their effectiveness and prognosis are typically less favorable, leading to high rates of recurrences and mortality [2, 3]. Nevertheless, targeted molecular therapy has rapidly developed in recent years, as evidenced by a growing number of studies exploring novel promising molecular therapeutic targets for glioma [4–6]. With a length of more than 200 nucleotides, long non-coding RNAs (lncRNAs) have been shown to regulate numerous biological processes [7, 8]. LncRNA nuclear paraspeckle assembly transcript 1 (NEAT1) is an essential component of the nuclear paraspeckle substructure [9]. Particularly, NEAT1 is a pro-oncogenic factor that plays a positive role in various solid tumors, such as liver, prostate, and gastric cancers, as well as renal cell carcinomas and glioma [10–14]. In glioma, NEAT1 has been found to regulate tumor progression via mTOR signaling pathway [15]. In fact, its downregulation reportedly inhibited glioma cell migration and invasion by regulating SOX2 [16]. Additionally, NEAT1 was shown to partially mediate TMZ resistance in glioma stem cells [17].

MicroRNAs (miRNAs) are a group of small non-coding RNAs comprising 18–24 base pairs with single-chain molecules. MiRNAs regulation, which is basically dependent on their interaction with mRNA 3'-untranslated region (3'-UTR) [18], has attracted numerous research attentions. For instance, exosomal miR-128-3p was found to facilitate chemosensitivity of colorectal cancer to oxaliplatin [19]. Besides, it has been reported that the malignant behavior of basal-like breast cancer cell lines was negatively regulated by miR-128-3p [20]. In glioma cells, miR-128-3p promoted apoptosis and conduced to mitochondrial dysfunction [21]; however, the underlying mechanisms of miR-128-3p function remain unclear and certainly worth further exploring.

Integrin family, a class of cell-surface-adhesion receptors that are formed by two noncovalently related subunits, namely α and β, reportedly regulate occurrence and development of tumor cells by mediating bidirectional signal transduction between cells and the extracellular matrix (ECM) as well as activating various intracellular signaling pathways [22–24]. An important subtype of the integrin α chain family, ITGA5, forms heterodimers together with integrin β1 (ITGB1). Previous studies have shown that ITGA5 is overexpressed in various cancer types, where it is involved in tumor progression [25–28]. Although its role in glioma has also been reported [29–31], the corresponding upstream and downstream mechanisms of action have not been fully clarified.

Although abnormalities of NEAT1, miR-128-3p, or ITGA5 expression have been reported in various studies about glioma, their potential interaction remains a puzzle. In this research, we hypothesized that the carcinogenesis of NEAT1 was regulated by the miR-128-3p/ITGA5 axis. Therefore, we analyzed their patterns of expression and biological functions in glioma, and elucidated the relationship among these factors with a view of providing useful insights to guide future development of treatment therapies for glioma.

## Methods and Materials

### Clinical Samples and Cell Lines

At least 30 specimens, comprising 10 grade I–II and 10 grade III–IV glioma tissues, as well as 10 normal brain tissues, were collected from patients who underwent surgical operation before anti-tumor treatments at Jinling Hospital. Non-tumorous brain tissues were extracted from the tumor border or normal areas of brain tissues removed from glioma patients. Before inclusion in the study, a written informed consent had been required from all patients. The study strictly complied with the guidelines of the Declaration of Helsinki, while ethical approval was obtained from the Ethics Committee of Jinling Hospital.

Human GBM cell lines, namely U87MG, A172, and U251MG, were purchased from the Cell Bank of Type Culture Collection of the Chinese Academy of Sciences (Shanghai, China), U118MG was obtained from the American Type Culture Collection (ATCC), while the human astrocyte cell lines (HA) were provided by the Institute of Basic Medical Sciences (Beijing, China). Cell lines were cultured in Dulbecco’s modified Eagle’s medium (DMEM), supplemented with 1% penicillin/streptomycin (HyClone, GE Healthcare Life Sciences, Logan, UT, USA) and 10% fetal bovine serum (Thermo Fisher Scientific, Waltham, MA, USA), and then maintained in a humidified incubator at 37°C with 5% CO2.

### Antibodies

Anti-integrin alpha 5 (ab150361) and anti-AGO2 (ab186733) antibodies were bought from Abcam (Cambridge, MA, USA). Tubulin β (AP0064) antibody was acquired from Bioworld Technology (MN, USA). Anti-phospho-FAK (#8556), anti-FAK (#3285), and anti-caspase-3 (#9662) were purchased from Cell Signaling Technology (Danvers, MA, USA), while anti-cyclin D1 (AF0931) was acquired from Affbiotech (Cincinnati, OH, USA).

### Cell Transfections

NEAT1 short hairpin RNA (shRNA) or hsa-miR-128-3p sponge was cloned into lentivirus vectors containing a GFP reporter gene to downregulate NEAT1 and miR-128-3p. A scramble sequence was used as control (shCtrl). Lentivirus vectors carrying ITGA5 sequence (NM_002205) or stem-loop sequence of hsa-miR-128-1 (MI0000447) were also constructed to overexpress ITGA5 and miR-128-3p, with an empty vector included as a negative control (pLV-Ctrl). Lentivirus vectors were constructed in Hanbio Biotechnology (Shanghai, China), and the transfection was performed according to the manufacturer’s recommended protocol. Cells were transfected with lentivirus for 48 h and selected by puromycin resistance.

### Cell Growth Assay

Cell viability was monitored using CCK-8 assay (Dojindo, Kumamoto, Japan), according to the manufacturer’s instructions. Briefly, cells were plated into 96-well plates at a density of 2000 cells/well, and their absorbance was measured at 450 nm using a Bio-Rad microplate reader. Measurements were done every 24 h after incubating the cells with CCK-8 reagent (at a 1:10 dilution) for 2 h.

### Transwell Assay

Transwell analysis was performed to detect motility of GBM cells, including migration and invasion assay. Briefly, approximately 1 × 10^5^ cells in 100-μL serum-free medium were added into the upper chamber of transwells (Costar, Corning, NY, USA) using a polycarbonate membrane (in diameter and pore size of 6.5 mm and 8 μm, respectively). Especially for the invasion assay, chambers were pre-coated with Matrigel (BD Biosciences, USA) the night before. Afterwards, 500 ml of DMEM supplemented with 10% FBS was added to the lower chamber. The contents were incubated for 24 h at 37°C, and then the Matrigel and cells in the top chambers were removed using cotton swab. Cells were fixed on the chambers with 4% paraformaldehyde for 20min and stained with 0.1% crystal violet for 20min. The stained cells were counted in 5 randomly selected microscopic fields (× 200) and the average number was recorded.

### Flow Cytometric Analysis

To assess the cell cycle, cells were harvested and fixed with 75% alcohol overnight, and then stained with propidium iodide (BD Biosciences, USA) in the dark. The stained cells were analyzed via the FACSCalibur flow cytometer (BD Biosciences, USA), and the percentage of cells at the G1/G0, S, and G2/M phases was counted using the ModFit software (Verity Software House, USA).

To analyze apoptosis, cells were stained with Annexin V-APC/7-AAD apoptosis detection kit (KGA1024, Jiangsu, China) according to the manufacturer’s recommendation, and then apoptotic cells were quantified on the FACScan system.

### Quantitative Real-Time PCR (qRT-PCR)

Total RNA was isolated from the aforementioned samples and cultured cells using Trizol reagent (Invitrogen, Carlsbad, CA, USA), which was then reverse transcribed into complementary DNA (cDNA) utilizing the first strand cDNA synthesis kit (Servicebio, China). The cDNA was used for qRT-PCR targeting the following genes: NEAT1, 5′-CTCTAGGTTTGGCGCTAAACTCTT-3′ and 5′-CCACCATTACCAACAATACCGACT-3′; miR-128-3p, 5′-CTCAACTGGTGTCGTGGAGTCGGCAATTCAGTTGAGAAAGAGAC-3′ and 5′-ACACTCCAGCTGGGTCACAGTGAACCGGT-3′; ITGA5, 5′-CTCCACAGATAACTTCACCCGA-3′ and 5′-GGCCTTGCCAGAAATAGCTT-3′; GAPDH, 5′-GGAAGCTTGTCATCAATGGAAATC-3′ and 5′-TGATGACCCTTTTGGCTCCCTGATGACCCTTTTGGCTCCC-3′; U6, 5′-CTCGCTTCGGCAGCACACTCGCTTCGGCAGCACA-3′ and 5′-AACGCTTCACGAATTTGCGT-3′. GAPDH was included as an internal amplification control for NEAT1 and ITGA5, while U6 was used as a control for miR-128-3p. Relative quantification was determined via the 2^-∆∆CT^ method.

### Western Blot Assay

Cells were lysed in RIPA buffer, containing with 1% PMSF, and the extracted proteins were quantified using the BCA method. Proteins were separated via SDS-PAGE and transferred onto polyvinylidene difluoride (PVDF) membranes (EMD Millipore, Billerica, MA, USA). The membranes were blocked with 5% nonfat milk for 2 h and incubated with specific primary antibodies overnight at 4°C. The membranes were washed in TBST, and then incubated with secondary antibodies at room temperature for 2 h. Finally, protein bands were exposed to a chemiluminescence imaging system (Tanon, Shanghai, China) with enhanced chemiluminescence detection reagents (EMD Millipore). The quantifications were evaluated using Image J software.

### Fluorescence In Situ Hybridization (FISH)

FAM-labeled probe for detecting miR-128-3p was synthesized by Servicebio (China). U87MG and U251MG cell climbing slices were fixed in 4% paraformaldehyde for 20 min and washed 3 times with PBS. Ulteriorly, the cells were incubated with pre-hybridization solution at 37°C for 1 h. The solution was removed followed by overnight incubation with probe hybridization solution with concentration of miR-128-3p (U6 and 18S were set as internal controls) in a humidified chamber at 42°C for hybridization. Subsequently, the cells were washed with 2 × saline-sodium citrate (SSC) for 10 min, 1 × SSC for 5 min, and 0.5 × SSC for 10 min at 37°C. Cell nuclei were stained with DAPI, which was applied for nuclear localization. The sequence for the miR-128-3p probe was as follows: 5′-DIG-AAAGAGACCGGTTCACTGTGA-3′.

### Dual-Luciferase Reporter Assay

Wild-type and mutant NEAT1 or ITGA5 genes were subcloned into psi-CHECK2 vectors (Promega). Whereafter, the vectors were co-transfected into 293T cells alongside miR-128-3p mimics or negative controls. Luciferase activities were measured after 48 h and normalized to those of Rluc, using the Promega Dual Luciferase Reporter system (Promega) according to the manufacturer’s recommendation.

### RNA Pull-Down Assay

Wild-type and mutant miR-128-3p or control RNA (NC) were biotin-labeled using the Pierce™ RNA 3' End Desthiobiotinylation Kit (Thermo Fisher) and incubated with magnetic beads (Thermo Fisher). The bead-biotin mixture was vigorously mixed prior to addition of the complex in U87MG cell lysates, and then incubated for 2 h with slow shaking. After washing with cell lysis buffer A, the RNAs that had been bound to the beads were captured, and relative levels of NEAT1 expression were assessed via qRT-PCR.

### RNA Immunoprecipitation Assay (RIP)

RIP assay was performed using the Magna RIP kit (Millipore) according to the manufacturer’s instructions. Summarily, U87MG cells transfected with pre-miR-128-3p or pLV-Ctrl were lysed in RIPA buffer, incubated overnight with anti-AGO2 antibody or IgG (served as a negative control), followed by conjugation with protein A + G agarose to capture the antigen-antibody complex. Finally, the levels of NEAT1 and miR-128-3p expression in the complex were determined by qRT-PCR.

### Tumor Heterograft Study

Experimental procedures involving animals were approved by the Animal Ethics Committee of Jinling Hospital. Subcutaneous xenograft GBM model was induced in BALB/c nude mice (4-week-old, male). Briefly, each nude mouse was injected in the right flank area with 5 × 10^6^ U87MG cells. Since the 8th day of modeling, tumor volumes were calculated every 4 days, and the formula was formulated as follow: volume (mm^3^) = (major axis) × (minor axis)^2^/2.

### Immunofluorescence (IF) Staining and TUNEL Staining

Mice were sacrificed 28 days after inoculation, tumor tissues collected were fixed with 4% paraformaldehyde for paraffin sections. Sections were subjected to immunofluorescence staining for Ki-67 (Cell Signal Technology, 1:200 dilution), as well as TUNEL assay with an in situ cell death detection kit (TMR red, Roche), according to the manufacturer’s protocol. Slides were observed under a Zeiss immunofluorescence microscope (Zeiss, Germany).

### Statistical Analysis

Statistical analyses were conducted using GraphPad Prism software version 5 (GraphPad Software, Inc., San Diego, CA, USA), and all data (from three independently experiments) presented as means ± standard deviations (SD). Comparisons between 2 groups were made using Student’s *t* test, and ANOVA was utilized for multiple group comparisons. Data followed by *P*<0.05 were considered statistically significant.

## Results

### NEAT1 Was Upregulated While miR-128-3p Was Downregulated in Human Glioma Tissues and Cell Lines

**Fig. 1:**
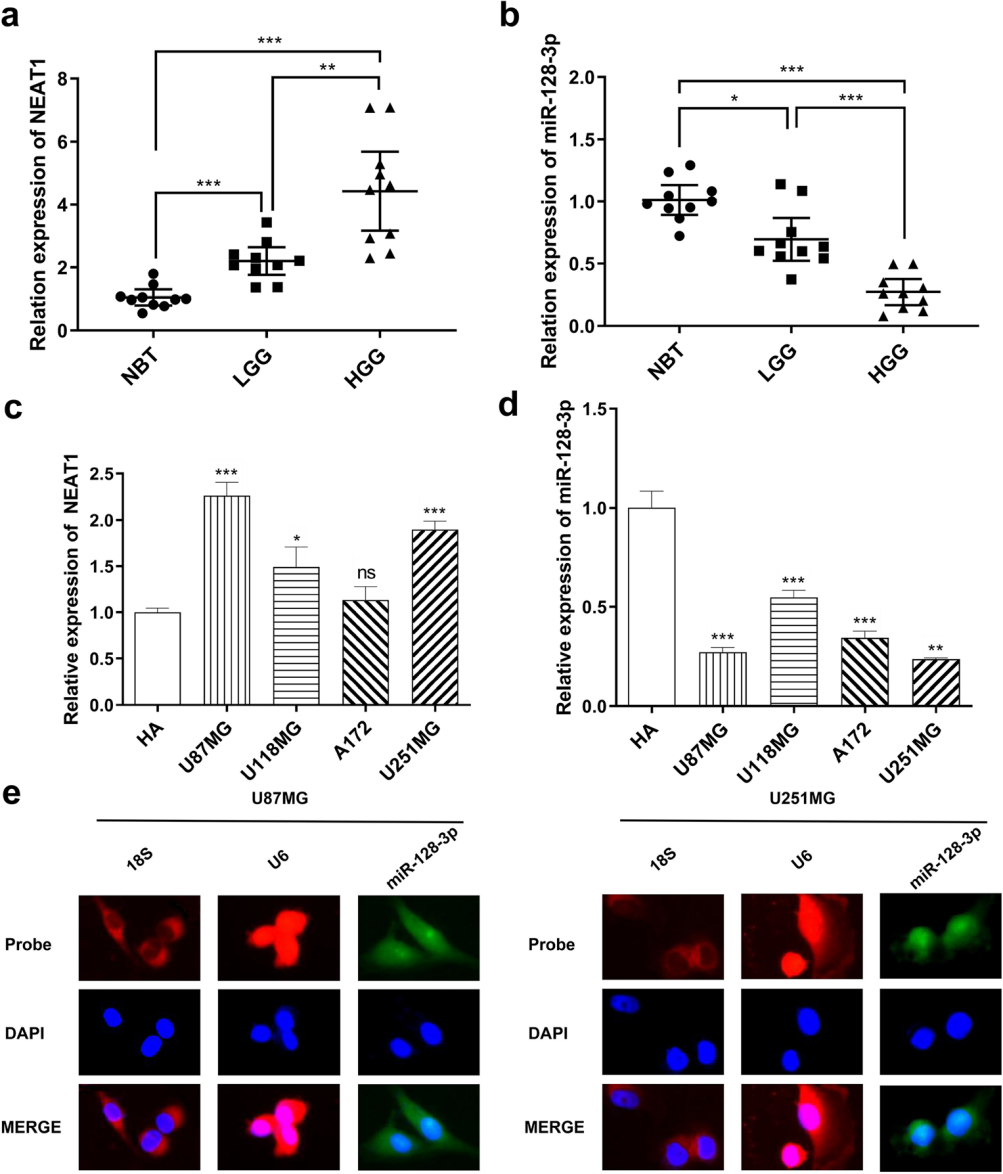
In glioma tissues and GBM cell lines, NEAT1 was upregulated whereas miR-128-3p showed an obvious decrease, and miR-128-3p was localized in both the nucleus and cytoplasm. **a, b** qRT-PCR showing NEAT1 and miR-128-3p expression levels in normal brain tissues (NBT) and different grades of glioma tissues (LGG, HGG), each group had 10 samples. *p < 0.05, **p < 0.01, ***p < 0.001. **c, d** Expression of NEAT1 and miR-128-3p in normal human astrocytes (HA) and GBM cell lines (U87MG, U118MG, A172, U251MG). *p < 0.05, **p < 0.01, ***p < 0.001 VS HA. **e** FISH assay verified the subcellular localization of miR-128-3p

To confirm whether NEAT1 and miR-128-3p expression was differentially expressed between normal brain tissues and glioma tissues, we performed qRT-PCR to assess NEAT1 and miR-128-3p expression in grade I–II glioma tissues (LGG) and grade III–IV glioma tissues (HGG), alongside normal brain tissues (NBT); ten cases were selected randomly for each group. As expected, NEAT1 expression levels elevated with the ascending of pathological grades of glioma tissues whereas miR-128-3p showed an opposite trend (Fig. 1a and b).

We validated these results by analyzing expression levels of NEAT1 and miR-128-3p in human astrocyte (HA) and a variety of human GBM cell lines, including U87MG, U118MG, A172 and U251MG. The results showed that NEAT1 was dramatically increased in glioma cell lines (except A172) relative to HA, especially in U87MG and U251MG (Fig. 1c). Similarly, miR-128-3p showed contrary results and was downregulated in glioma cells (Fig. 1d).

### MiR-128-3p Was Directly Regulated by NEAT1

Accumulating studies have proved that lncRNAs could be competing endogenous RNAs (ceRNAs) or molecular sponges, thereby jeopardizing expression and biological functions of specific miRNAs in the cytoplasm [32]. Through the starBase prediction software (http://starbase.sysu.edu.cn/), we found that the 3′UTR of NEAT1 contained a potential miR-128-3p binding site. However, NEAT1 was localized in the nucleus instead of the perinuclear region. On the other hand, several studies have also verified that miRNAs can target nuclear lncRNAs, which requires that the miRNAs should be distributed in the nucleus [33, 34]. Based on this, we employed FISH to analyze subcellular localization of miR-128-3p. Just as showed in Fig. 1e and f, it was present both in the nucleus and cytoplasm.

**Fig. 2:**
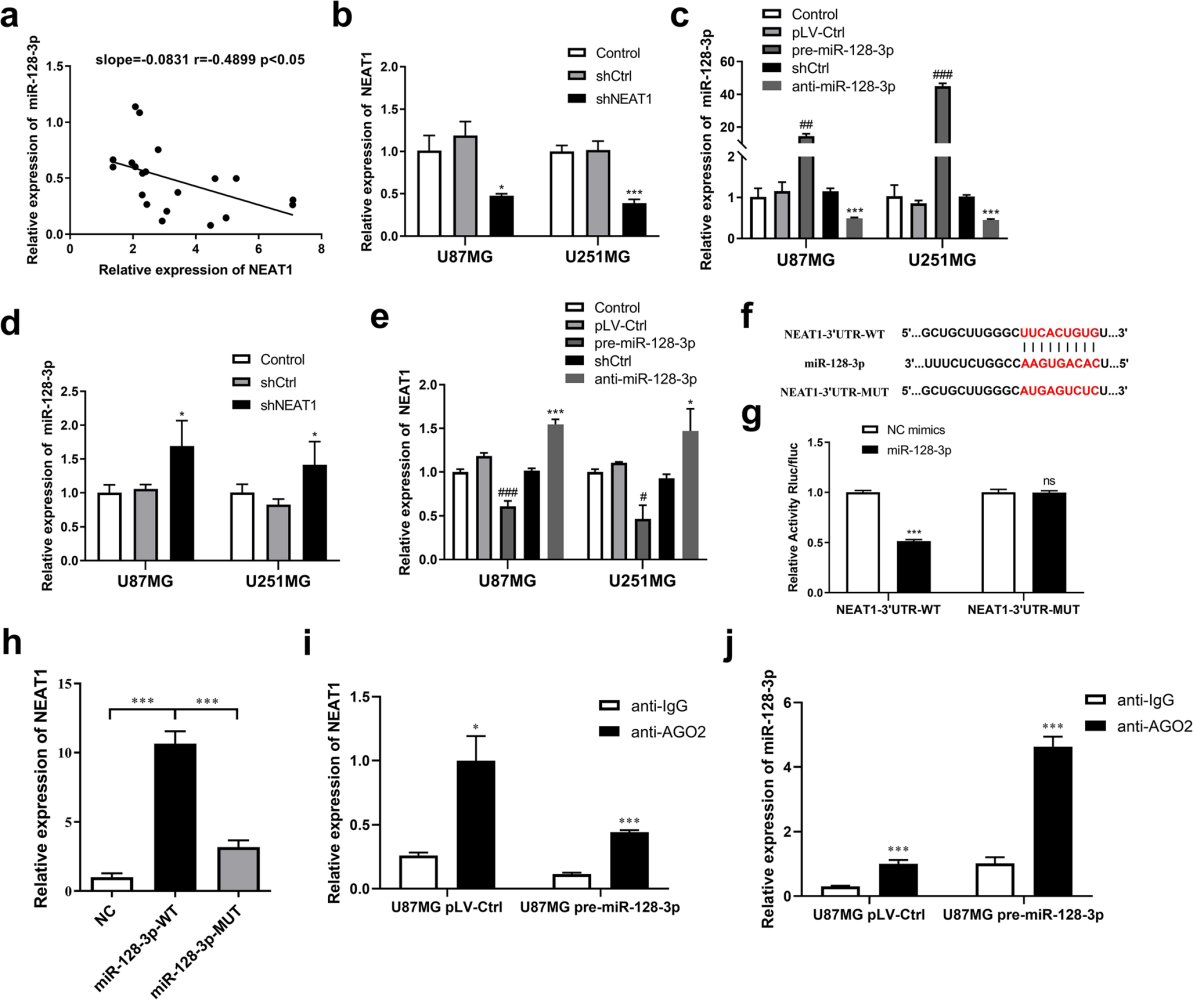
NEAT1 was a direct target gene of miR-128-3p. **a** Linear regression analysis between expression levels of NEAT1 and miR-128-3p in glioma tissues. **b–e** Effects of lentiviral transfections were confirmed by qRT-PCR. An inversely relationship was observed between expression levels of NEAT1 and miR-128-3p in glioma cells. *p < 0.05, ***p < 0.001 VS shCtrl group; #*p* < 0.05, ##*p* < 0.01, ###*p* < 0.001 VS pLV-Ctrl group. **f** The hypothesized wild-type or mutational miR-128-3p binding sites in ITGA5. **g** Luciferase activities in U87MG cells co-transfected with vector NEAT1-3′UTR-WT or NEAT1-3′UTR-MUT and miR-128-3p mimics or NC mimics was analyzed by Luciferase reporter assay. ***p < 0.001. **h** Specific association between NEAT1 and miR-128-3p following a RNA pull-down assay in U87MG cells. ***p < 0.001. **i, j** RIP assay indicating both of NEAT1 and miR-128-3p were enriched by anti-AGO2 in U87MG cells, whether miR-128-3p was overexpressed beforehand or not. *p < 0.05, ***p < 0.001 VS anti-IgG group

Meanwhile, correlation analysis revealed that expression of NEAT1 was negatively associated with that of miR-128-3p in human glioma tissues (Fig. 2a). To further verify this association, we stably knocked down NEAT1 (shNEAT1) and miR-128-3p (anti-miR-128-3p), as well as overexpressed miR-128-3p (pre-miR-128-3p), by transfecting lentivirus with U87MG and U251MG. Efficiency of the transfections was confirmed via qRT-PCR (Fig. 2b and c). We next detected upregulation of miR-128-3p in the shNEAT1 group comparing with the shCtrl group (Fig. 2d). In addition, NEAT1 was also reduced and increased in the pre-miR-128-3p and anti-miR-128-3p groups, respectively (Fig. 2e). Moreover, results of the RNA pull-down assay demonstrated that NEAT1 was significantly enriched in the miR-128-3p-WT group, relative to the NC and miR-128-3p-MUT groups (Fig. 2h). Furthermore, luciferase reporter assay was conducted to validate the predicted binding sites for NEAT1 and miR-128-3p. MiR-128-3p mimics downregulated NEAT1-3′UTR-WT activity, but this was not the case in NEAT1-3′UTR-MUT (Fig. 2f and g).

It has also been proven that mature miRNAs are preferentially loaded onto the RNA-induced silencing complex (RISC), which contains AGO2, a core protein of RISC [35]. For this reason, RIP assay using antibody AGO2 was performed in U87MG cells to confirm whether NEAT1 suppressed miR-128-3p function by interacting with AGO2 in the nucleus. As shown in Fig. 2i and j, both NEAT1 and miR-128-3p were sufficiently enriched by anti-AGO2 rather than anti-IgG, whether they were in the pLV-Ctrl group or pre-miR-128-3p group.

To sum up, miR-128-3p was directly regulated by NEAT1, suggesting existence of a regulatory feedback loop between both factors.

### NEAT1 Knockdown Suppressed Tumor Progression in GBM Cell Lines by Regulation of miR-128-3p

**Fig. 3:**
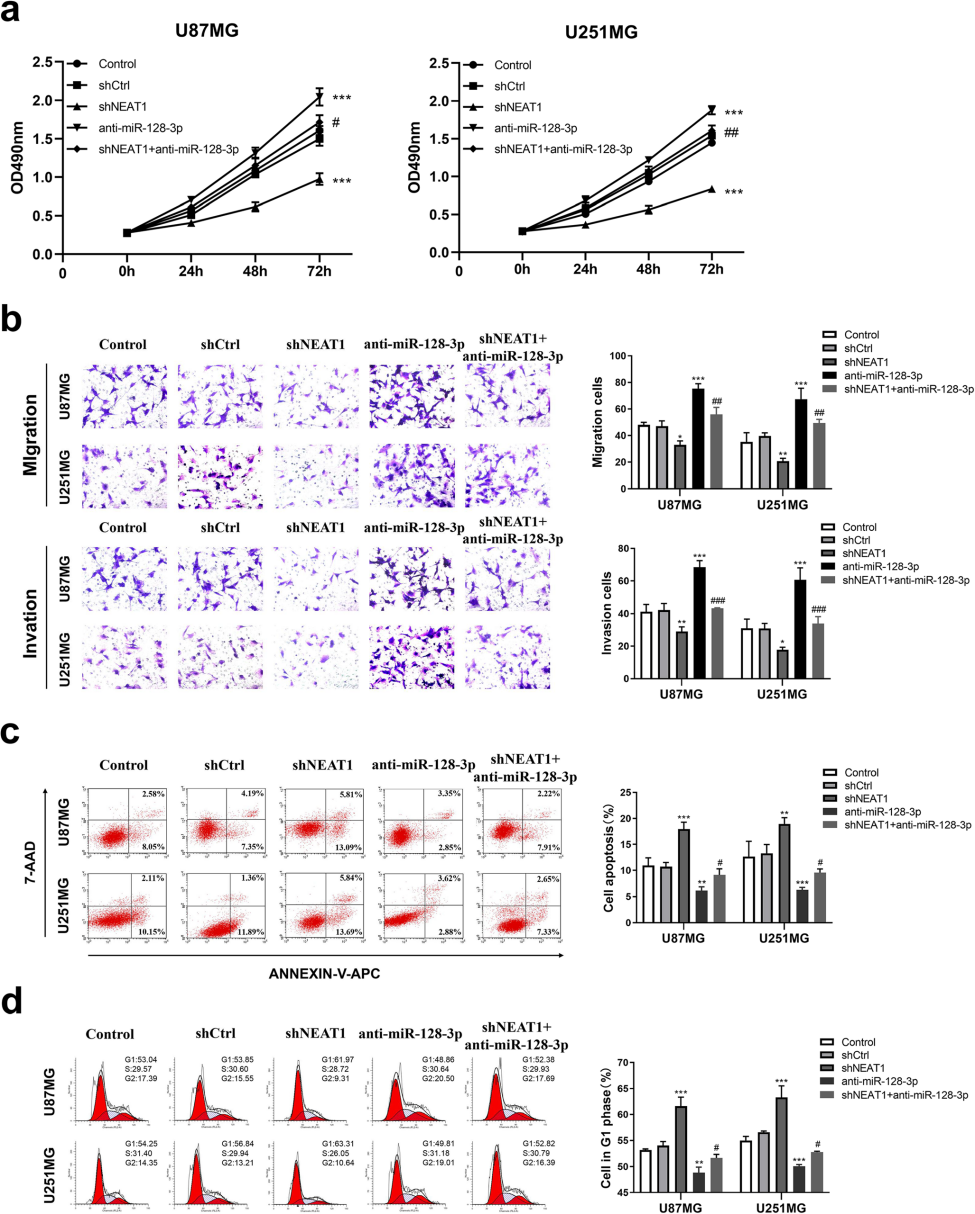
NEAT1/miR-128-3p axis participated in glioma progression in vitro. **a** CCK-8 assays were carried out to appraise the effects of NEAT1 and miR-128-3p on cell viability in U87MG and U251MG cells. **b** Transwell assays were applied to measure the effects of NEAT1 and miR-128-3p on cell viability in U87MG and U251MG cells. **c, d** Flow cytometry were employed to evaluate the effects of NEAT1 and miR-128-3p on cell apoptosis and cell cycle in U87MG and U251MG cells. **p < 0.01, ***p < 0.001 VS shCtrl group; #*p* < 0.05, ##*p* < 0.01, ###*p* < 0.001 VS anti-miR-128-3p group

To assess whether NEAT1 disrupts tumor progression via regulating miR-128-3p, U87MG and U251MG stably expressing anti-miR-128-3p were co-transfected with shNEAT1. Afterwards, the cell lines were divided into 5 groups, namely control, shCtrl, shNEAT1, anti-miR-128-3p, and shNEAT1+anti-miR-128-3p. CCK-8 assays revealed considerably higher growth rates in the anti-miR-128-3p group than the shCtrl and shNEAT1+anti-miR-128-3p groups, in both types of GBM cells. Conversely, cell proliferation was diminished substantially in the shNEAT1 group (Fig. 3a). Transwell assays showed a dramatic increase in cell migration and invasiveness in the anti-miR-128-3p group, relative to the shCtrl and shNEAT1+anti-miR-128-3p groups, but the shNEAT1 group exhibited a weaker migrative and invasive capacity (Fig. 3b). Subsequently, as measured by flow cytometry, the shNEAT1 group had higher apoptosis rates, while those in the anti-miR-128-3p group was notably lower compared to the shCtrl and shNEAT1+anti-miR-128-3p groups (Fig. 3c). Moreover, the percentage of cells in G1 phase was apparently increased in the shNEAT1 group and decreased in the anti-miR-128-3p group, whereas the phenomenon was not obvious in the shNEAT1+anti-miR-128-3p group, which was consistent with the results above (Fig. 3d). Collectively, knockdown of NEAT1 reversed the biological functions of GBM cells which had low miR-128-3p expression. In consequence, miR-128-3p was implicated in the tumor-suppressive effects of NEAT1 knockdown in GBM cell lines.

### NEAT1 Combined with miR-128-3p Played an Integral Part in Glioma Growth In Vivo

**Fig. 4:**
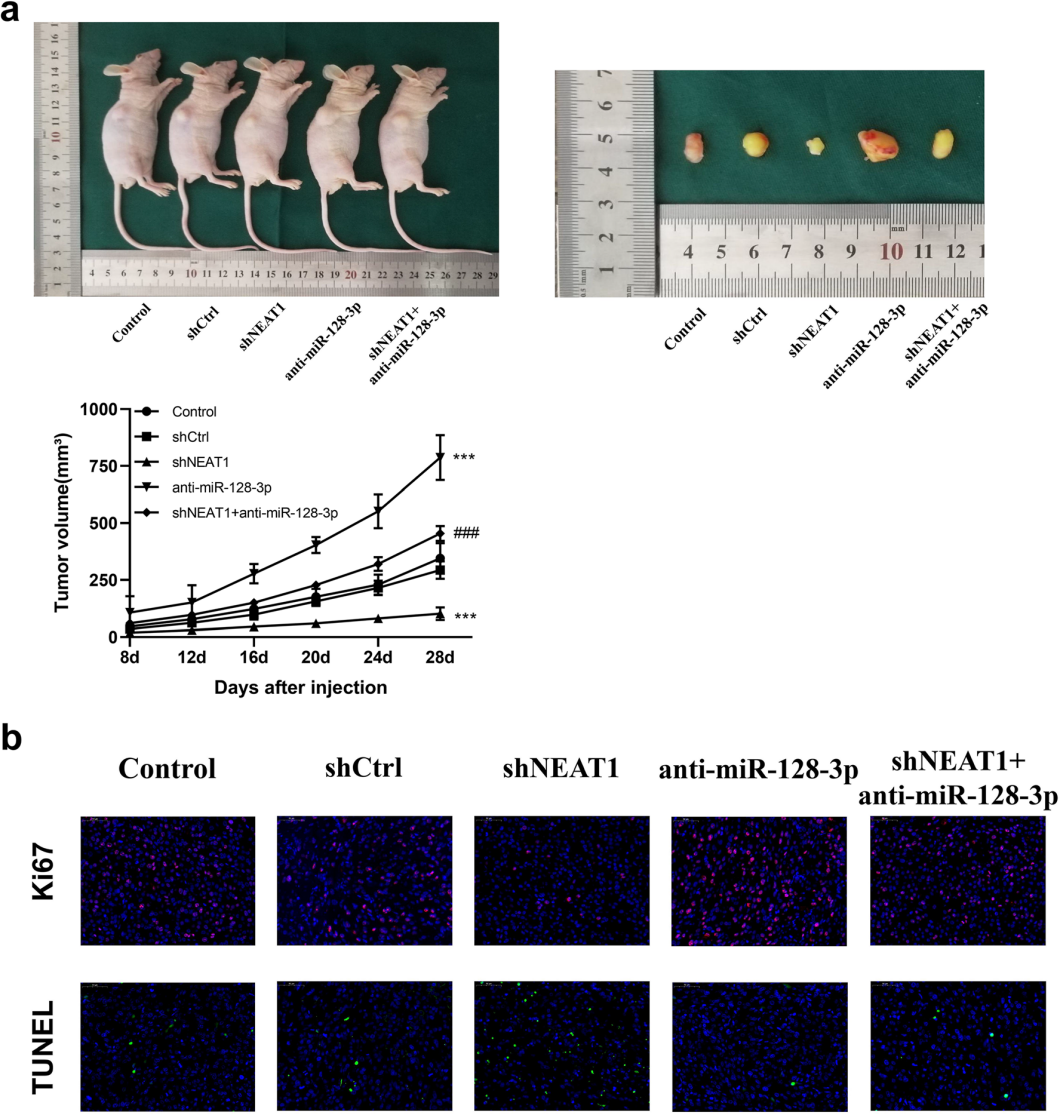
NEAT1/miR-128-3p axis was involved in glioma growth *in vivo*. **a** Subcutaneous xenotransplanted GBM model was established by intratumorally injecting U87MG cells into the right flank of nude mice. Tumors were harvested 28 days after injection, and tumor volume curves of nude mice in each group (*n* = 5) were plotted. ***p < 0.001 VS shCtrl group; ###*p* < 0.001 VS anti-miR-128-3p group. **b** IF staining of Ki-67 and TUNEL staining of tumor sections collected from nude mice were performed. Scale bar represents 50 mm

To further affirm the relevancy between NEAT1 and miR-128-3p on glioma growth in vivo, we established a tumor model in nude mice. Notably, NEAT1 knockdown delayed tumor growth in the model compared to the control group. Conversely, mice in the anti-miR-128-3p group manifested significantly larger tumor volumes than those in the shCtrl and shNEAT1+anti-miR-128-3p groups (Fig. 4a). Besides, results from IF staining corroborated those from CCK-8 assays and flow cytometry. Specifically, mice in the shNEAT1 and shNEAT1+anti-miR-128-3p groups had relatively lower positive rates of proliferation index Ki-67 than those in the anti-miR-128-3p group. Moreover, TUNEL staining revealed that mice in the former two groups had higher positivity rates comparing with the latter group (Fig. 4b). Overall, these results implied that NEAT1 promoted tumorigenesis by interacting with miR-128-3p both in vitro and in vivo.

### ITGA5 Was Overexpressed in Glioma Tissues and Regulated by the NEAT1/miR-128-3p Axis in Glioma Cell Lines

**Fig. 5:**
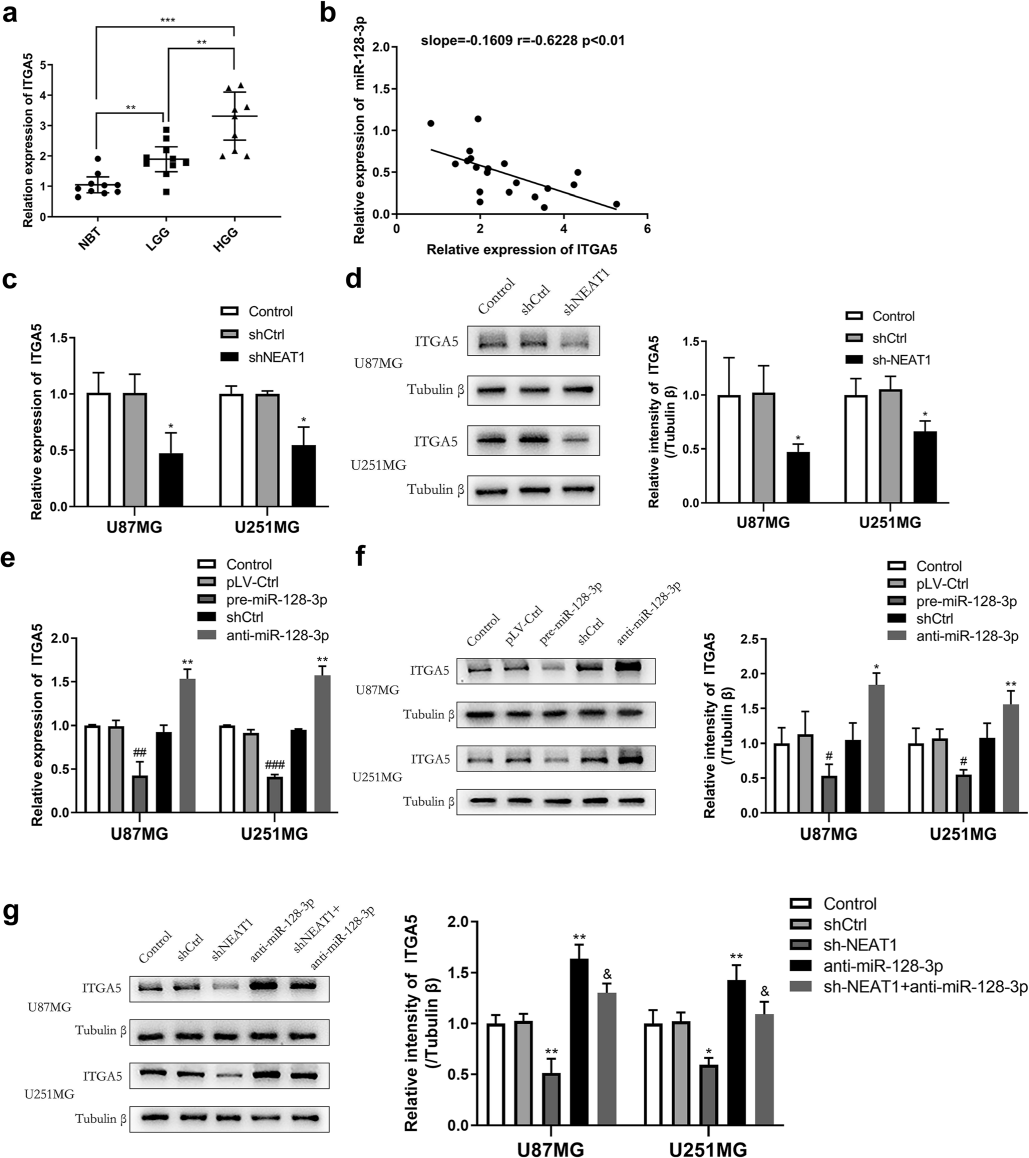
ITAG5 was an oncogene of glioma regulated by the NEAT1/miR-128-3p axis. **a** Profile of ITGA5 expression in glioma specimens following qRT-PCR. **b** Linear regression showing the relationship between expression levels of ITGA5 and miR-128-3p in glioma samples. **c–g** ITGA5 expression was assessed by qRT-PCR and western blot analysis in U87MG and U251MG cells transfected with various lentivirus about NEAT1 and miR-128-3p to explore the part of ITGA5 in them. *p < 0.05, **p < 0.01 VS shCtrl group; #*p* < 0.05, ##*p* < 0.01, ###*p* < 0.001 VS pLV-Ctrl group; & *p* < 0.05 VS anti-miR-128-3p group

To investigate the underlying molecular mechanisms of the NEAT1/miR-128-3p axis in modulating tumorigenesis of glioma, we predicted gene ITGA5 might be a downstream target of miR-128-3p according to the online database TargetScan (http://www.targetscan.org/vert_72/). Firstly, qRT-PCR was performed to analyze levels of ITGA5 expression in glioma samples; the results showed that it progressively increased from NBT to LGG and HGG, indicating a close correlation between ITGA5 and tumor grades (Fig. 5a). On the other hand, linear regression analysis revealed that expression of ITGA5 was inversely correlated with that of miR-128-3p in these tissues (Fig. 5b).

After that, we quantified mRNA and protein levels of ITGA5 by qRT-PCR and western blot analysis with the aim of understanding the impact of NEAT1 and miR-128-3p on this gene. As shown in Fig. 5c–f, ITGA5 expression was elevated upon downregulation of miR-128-3p, but it was reduced following NEAT1 knockdown. Above all, when we knocked down both miR-128-3p and NEAT1, ITGA5 expression levels remained normal (Fig. 5g), suggesting that NEAT1 might competitively bind miR-128-3p with ITGA5.

### MiR-128-3p Restrained Pro-tumorigenic Effects of ITGA5 by Targeting Its 3′UTR

**Fig. 6:**
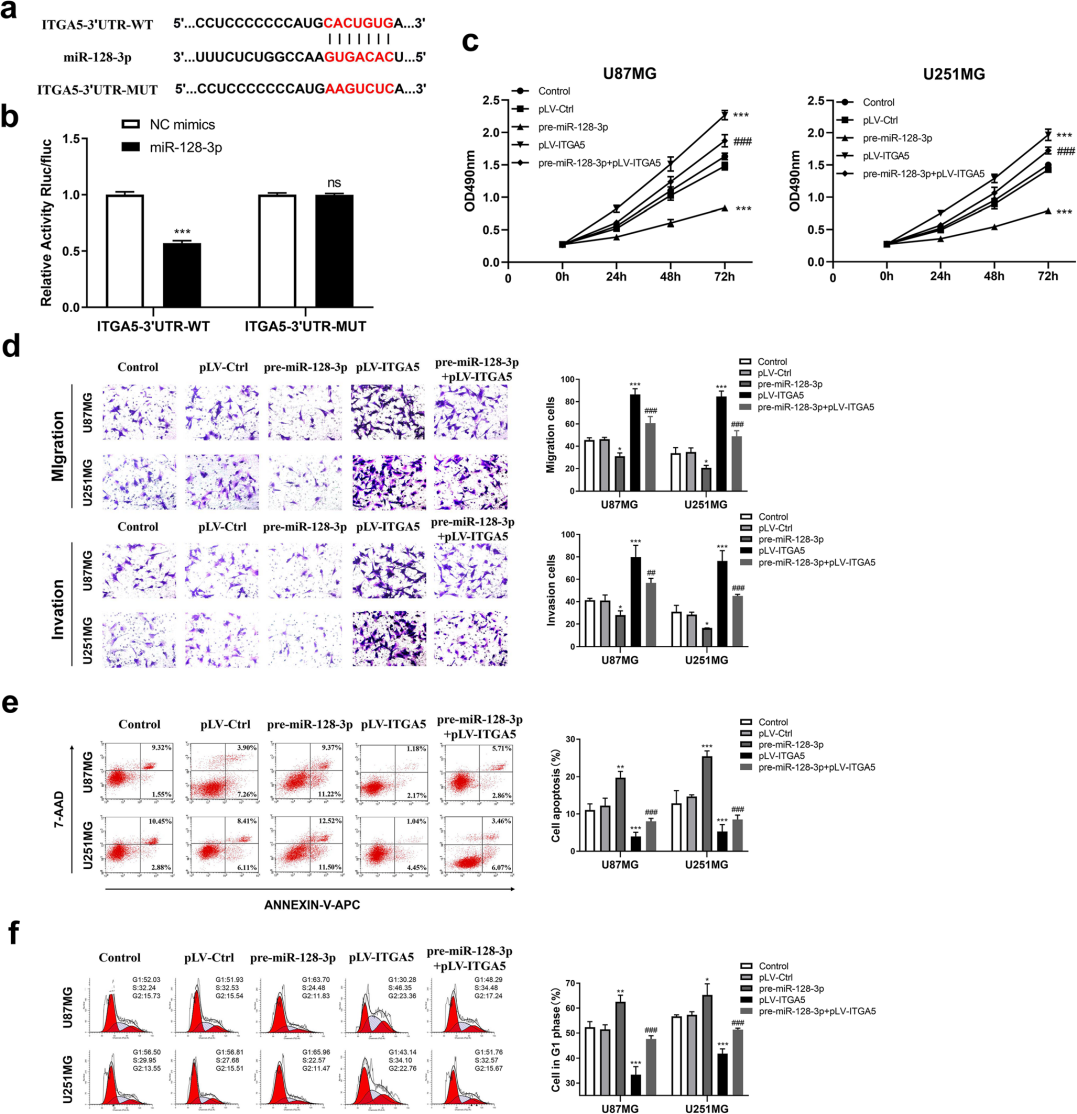
miR-128-3p mediated the pro-oncogenic effects of ITGA5. **a** The predicted wild-type or mutated miR-128-3p binding sites in ITGA5. **b** Luciferase activities in U87MG cells co-transfected with vector ITGA5-3′UTR-WT or ITGA5-3′UTR-MUT and miR-128-3p mimics or NC mimics as determined with luciferase reporter assay. ***p < 0.001. **c** The effects of NEAT1 and miR-128-3p on the proliferation of U87MG and U251MG cells as examined with CCK-8 assays. **d** The effects of NEAT1 and miR-128-3p on the migration and invasion of U87MG and U251MG cells as evaluated with transwell assays. **e, f** The effects of NEAT1 and miR-128-3p on apoptosis and cell cycle progression of U87MG and U251MG cells were determined by flow cytometry assays. *p < 0.05, **p < 0.01, ***p < 0.001 VS pLV-Ctrl group; #*p* < 0.05, ##*p* < 0.01, ###*p* < 0.001 VS pre-miR-128-3p group

To figure out whether miR-128-3p specifically inhibits ITGA5 via its potential binding site of seed sequence, we conducted a luciferase assay. Results indicated that the fluorescence intensity of the ITGA5-3′UTR-WT and miR-128-3p mimics co-transfected group was highly reduced, while that of the ITGA5-3′UTR-MUT and miR-128-3p mimics co-transfected group showed no significant differences (Fig. 6a, b), suggesting the specific binding site of miR-128-3p in the ITGA5-3′UTR.

Subsequently, we evaluated the role played by ITGA5 with miR-128-3p in glioma cells with regard to cell proliferation, migration, invasion, apoptosis, and cell cycle of GBM cell lines. As presented in Fig. 6c, ITGA5 overexpression accelerated proliferation of cell lines, and partially rescued the impaired growth of cells induced by miR-128-3p overexpression alone. Similar results were obtained in transwell assays, where stronger migratory and invasive abilities were observed in cells from the ITGA5 overexpression group, relative to those in the control group and the miR-128-3p overexpression combined with ITGA5 overexpression group (Fig. 6d). Moreover, flow cytometry revealed that ITGA5 overexpression eliminated arrest of the G1 cell cycle and restrained cell apoptosis arising from miR-128-3p overexpression (Fig. 6e and f). Overall, these results suggested that miR-128-3p could suppress tumor-promoting effects of ITGA5 in glioma cells.

### ITGA5 Played Its Oncogenic Role via the FAK Signaling Pathway

**Fig. 7:**
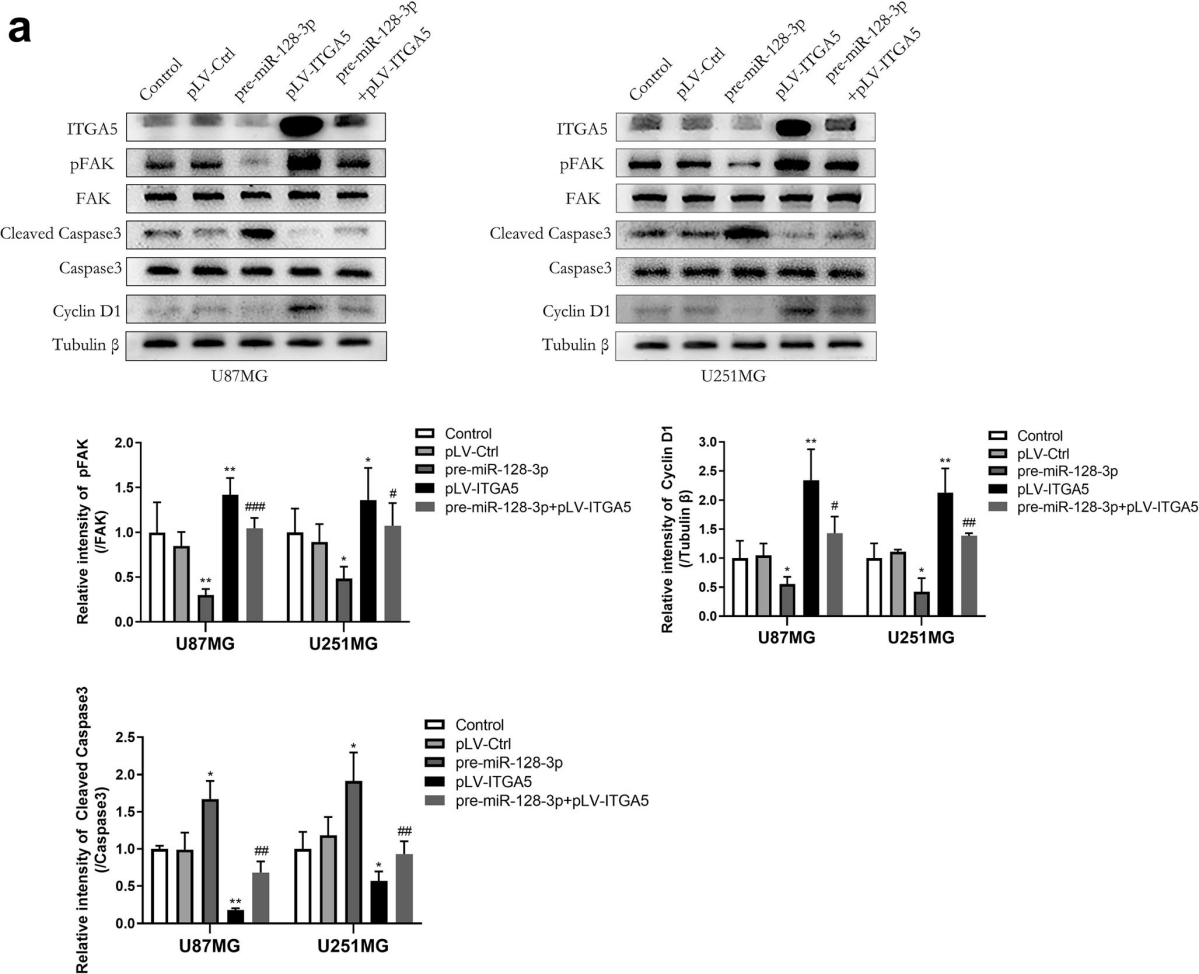
ITGA5 activated FAK signaling pathway in glioma cells. **a** Expression levels of the FAK signaling pathway related proteins such as phosphorylated and non-phosphorylated forms of FAK, total and cleaved caspase-3 as well as cyclin D1 were quantified by western blotting assays. *p < 0.05, **p < 0.01 VS pLV-Ctrl group; #*p* < 0.05, ##*p* < 0.01 VS pre-miR-128-3p group

Given the fact that focal adhesion kinase (FAK) is a heavily expressed nonreceptor protein-tyrosine kinase involved in integrin-mediated signaling pathways [36], we therefore evaluated protein levels of tyrosine phosphorylation of FAK in glioma cells via western blot assays, which could represent cell motility [37]. We also separately analyzed protein levels for FAK downstream genes, such as caspase-3 and cyclin D1, which directly regulate apoptosis and the G1 phase transition, respectively. Results showed that ITGA5 prompted phosphorylation of FAK and expression of cyclin D1, and this was accompanied by suppression of the transformation of caspase-3 to cleaved caspase-3 (Fig. 7a). Cells transfected with pre-miR-128-3p, which led to deletion of ITGA5, exhibited a completely opposite expression pattern. These results corroborated our previous findings, indicating that the FAK signal transduction pathway participated in the pro-tumorigenic effects of ITGA5 in glioma cells.

## Discussion

Although glioma is the most frequent primary malignant brain neoplasm, its prognosis remains relatively poor [38]. To date, numerous research efforts have enabled development of targeted molecular therapeutics for glioma, though the resulting clinical implications and efficiency remain unsatisfactory [39], necessitating explanation of the underlying molecular mechanisms of the disease. Results of the present work revealed upregulation of NEAT1 and downregulation of miR-128-3p in surgical glioma tissues and GBM cell lines, respectively. Furthermore, it was evident that NEAT1 could promote tumorigenesis by directly regulating miR-128-3p both in vitro and in vivo. ITGA5 was subsequently identified to interact with the NEAT1/miR-128-3p axis, and NEAT1 safeguarded the cancer-promoting effects of ITGA5 by binding to miR-128-3p competitively. Herein, the oncogenic role of ITGA5 was partially executed via the FAK signaling pathway.

LncRNAs have attracted accumulating research attention owing to their crucial impact on multitudinous malignant tumors recent years [40]. Our results revealed that lncRNA NEAT1 was aberrantly upregulated in GBM cell lines and was directly associated with glioma grades, which was consistent with recent studies [41, 42], indicating its tumorigenicity in glioma. On the other hand, intranuclear lncRNAs have been shown to play various functional roles at the post-transcriptional, transcriptional, and epigenetic levels during gene expression. Particularly, they constitute a functionally interactive network with chromatin, proteins, and other RNA molecules within the nuclear environment [9]. To further determine the possible molecular mechanism, we used the starBase prediction software and miR-128-3p was suspected to be related to NEAT1 by binding its 3′UTR. Although NEAT1 is primarily localized in the nucleus, past studies have demonstrated that miRNAs, which exist both within and outside the nucleus, can combine with nuclear lncRNAs. For example, lncRNA metastasis-associated lung adenocarcinoma transcript 1 (MALAT1) was found to target miR-9 in the nucleus [34]. Coincidentally, Liu et al. [43] showed that NEAT1 with miR-124-3p regulatory network contributed to proliferation of hepatocellular carcinoma.

According to the literature, the tumor-suppressive effects of miR-128-3p in tumors are evident, especially in glioma [44, 45]. Results of the present study indicated that miR-128-3p was decreased markedly in glioma tissues and cell lines. Strikingly, apart from cytoplasmic localization, miR-128-3p was abundant in the nucleus, suggesting that miR-128-3p could be interacting with NEAT1 in the nucleus. Notably, knocking down both factors upregulated expression of the other reciprocally, thereby confirming this association. This point was affirmed by the results from the RNA pull-down assay, which revealed significant enrichment of NEAT1 by the miR-128-3p probe. To gain insights into the mechanisms of interaction, luciferase reporter and RIP assays were performed. Overall, RISC consisted of miR-128-3p and AGO2 would regulate NEAT1 expression by couple its 3′UTR. Results of cell viability, motility, apoptosis, and cycle affirmed that NEAT1 was indeed a pro-oncogenic factor whereas miR-128-3p functioned as a tumor suppressor miRNA in glioma cells. Above all, NEAT1 inhibition impaired the high degree of malignancy of glioma cells triggered by the reduction of miR-128-3p. Analogously, in contrast to miR-128-3p downregulation alone, co-downregulation of NEAT1 and miR-128-3p in xenograft cells resulted in larger tumor volume. Moreover, immunofluorescence and TUNEL staining results manifested that bigger tumors had higher rates of Ki-67 positivity and lower rates of apoptosis. Taken together, these results showed that NEAT1’s tumorigenic properties resulted from adsorbing and disrupting miR-128-3p, and further exploration is required to validate this finding.

Database TargetScan predicted that 3′UTR of ITGA5 was conjectured to own the binding site of miR-128-3p, which was later experimentally validated by luciferase reporter assay. Interestingly, ITGA5 has been previously shown to be a tumor promoter in multiple tumor types, including glioma [46–48]. Coincidentally, ITGA5 was conspicuously upregulated in surgical specimens we collected but it was inversely correlated with miR-128-3p expression. Moreover, results from western blot and qRT-PCR analysis divulged that ITGA5 was regulated by NEAT1 together with miR-128-3p, and NEAT1 could sponge miR-128-3p with ITGA5 competitively. Furthermore, in vitro experiments revealed that overexpressing ITGA5 enhanced cell proliferation, migration, and invasion, but inhibited cell apoptosis and lessened G1 cell cycle arrest. Meanwhile, ITGA5 mitigated and restored miR-128-3p-induced adverse effects in glioma cell lines. These findings indicated that the biological functions of the NEAT1/miR-128-3p axis in glioma were dependent on the oncogene ITGA5. Further analysis revealed that the FAK signaling pathway was activated, and this was accompanied by upregulation of cyclin D1 which pushed cell cycle G1/S transition as well as less cleaved caspase-3 that mediated cell apoptosis. Besides, a significant phosphorylation of FAK was observed in cells overexpressing ITGA5, indicative of high migration and invasion of glioma cells.

## Conclusions

Although NEAT1, miR-128-3p, and ITGA5 have been widely implicated in various tumors, their relationship remains largely unknown in glioma. Our studies demonstrated the antioncogenic effects of miR-128-3p, as well as pro-oncogenic impacts of NEAT1 and ITGA5 in glioma cells. Notably, ITGA5 was depleted by miR-128-3p, whereas NEAT1 would forestall that and sponge miR-128-3p competitively so that ITGA5 could activate FAK signaling pathway and facilitate glioma progression. Taken together, our studies provide novel insights into the NEAT1/miR-128-3p/ITGA5 axis in glioma, and are expected to guide future development of therapies for glioma treatment.

## Data Availability

Data are available after the request.

## Abbreviations

Integrin α5: ITGA5
Argonaute-2: AGO2
CCK-8: Counting Kit-8
PCR: Polymerase chain reaction
